# Recurrence/Regrowth in Grade I Meningioma: How to Predict?

**DOI:** 10.3389/fonc.2020.01144

**Published:** 2020-08-04

**Authors:** Gervásio Teles Cardoso de Carvalho, Warley Carvalho da Silva-Martins, Kênia Cristina Soares Fonseca de Magalhães, Cristiana Buzelin Nunes, Aleida Nazareth Soares, Luciene Simões de Assis Tafuri, Renata Toscano Simões

**Affiliations:** ^1^Laboratory of Molecular Biology and Biomarkers, Santa Casa de Belo Horizonte Ensino e Pesquisa - EP/SCBH, Belo Horizonte, Brazil; ^2^Department of Neurosurgery, Hospital Santa Casa de Belo Horizonte, Belo Horizonte, Brazil; ^3^Faculdade de Ciências Médicas de Minas Gerais (FCMMG), Belo Horizonte, Brazil; ^4^Hospital Risoleta Tolentino Neves, Belo Horizonte, Brazil; ^5^Departamento de Anatomia Patológica e Medicina Legal, Universidade Federal de Minas Gerais, Belo Horizonte, Brazil; ^6^Centro Universitário de Belo Horizonte - UniBH, Belo Horizonte, Brazil; ^7^Departamento de Fisiologia e Patologia, Centro de Ciências da Saúde, Universidade Federal da Paraíba, DFP/CCS/UFPB, João Pessoa, Brazil

**Keywords:** immunomarkers, HLA-G, HLA-E, hormonal receptors, MIB-1, inflammatory biomarkers

## Abstract

The HLA-G and HLA-E molecules, Ki67, progesterone (PR), estrogen (ER) and androgen receptors (AR), p53, COX-2, and HER2 were studied to assess whether the biological behavior of grade I meningiomas is related to their expression. Tissue samples from 96 patients with grade I intracranial meningiomas were analyzed by immunohistochemistry on tissue microarray blocks (TMA) using antibodies specific for HLA-G, HLA-E, Ki67, PR, ER, AR, p53, COX-2, and HER2. Meningiomas were classified as small (≤2 cm, 1.0%), medium (>2 and ≤4 cm, 32.3%), and large (>4 cm, 66.7%). Tumor size was not related to recurrence/regrowth (*p* = 0.486), but was significantly correlated with peritumoral edema (*p* = 0.031) and intratumoral calcifications (*p* = 0.018). Recurrent meningiomas were observed in 14.6% of cases. Immunostaining for each marker was: HLA-G 100%; HLA-E 95.6%; PR 62%; ER 2.1%; AR 6.5%; p53 92.6%; COX-2 100%; HER2 0%; Ki67, mean 2.61 ± 2.29%, median 2.1%. Primary and recurrent meningiomas showed no significant relation with HLA-E and hormone receptors (*p* > 0.05), except for Ki67, where a higher median was observed in recurrent tumors than in primary (*p* = 0.014). The larger the tumor, the more severe the peritumoral edema, and the greater the presence of calcifications. Ki67 appears to be a good biomarker of recurrence/regrowth in grade I meningiomas.

## Introduction

Meningiomas originate from arachnoid cells, mainly from the villi (1). They correspond to 36.8% of intracranial primary tumors, affect females more than males, and their incidence increases greatly after age 65 (2). The World Health Organization report of 2016 classifies meningiomas as grades I, II and III (3). Even grade I tumors recur with frequency (4) from 7 to 20% (5), particularly in younger patients and males (6, 7).

The biological behavior of meningiomas cannot be predicted solely based on histopathology (8, 9). Thus, mitotic and cell proliferation indices (Ki67), the intensity of angiogenesis, the influence of sex steroid hormones (PR, ER, AR), inflammatory markers (COX-2), tumor suppressors (p53), genetic and immunological changes and histocompatibility molecules (HLA-G and HLA-E) can contribute for toward pathogenesis and the development of meningioma.

The predominance of meningiomas among females raises questions concerning the potential actions of sex hormone receptors in the pathogenesis and development of this type of tumor (10). The literature shows controversial data on the action of hormone receptors, such as progesterone (PR), estrogen (ER) and androgen (AR), particularly for PR in recurrent meningiomas (11–14). HLA-G and HLA-E are non-classical molecules belonging to the major histocompatibility complex (HLA) in humans and are located on the short arm of the chromosome 6 (15). HLA-G and HLA-E show immunosuppressive and tolerance-inducing functions, inhibiting the cytolytic activities of natural killer (NK) cells (16) and cytotoxic T and B lymphocytes (17). Physiologically, HLA-G and HLA-E are present in placental villi and protect the fetus against maternal antibodies (18). Through the same mechanism, these molecules can also protect tumors against the immune defense mechanisms of their carriers (19). The expression of these molecules has been demonstrated in numerous tumor types, and they are considered signs of malignancy (20), including in the central nervous system (CNS) tumors (21–23). A study by our group showed higher rates of soluble HLA-G expression in multiform glioblastomas than in meningiomas (24). To the best of our knowledge, no work on the expression of HLA-G and HLA-E in meningiomas has been published.

Ki67 is a nuclear protein associated with the mitotic activity that functions as a marker of the cell proliferation index (CPI) and is present in all phases of the cell cycle, except the G0 phase. This protein has been shown to be effective at determining the CPI in meningiomas (25) and has been routinely used in clinical practice (13, 26) because it is related to the mitotic index and histopathological grade in meningiomas (26, 27). Higher Ki67 expression in recurrent meningiomas than in primary patients has been previously described, and analysis of this marker is an important method for predicting early recurrence in meningiomas (27–29). In breast cancer, Ki67 is well established as a marker of prognosis and the responses to endocrinological treatment and chemotherapy (26).

Nuclear phosphoprotein p53 acts on the cell cycle, essentially in the maintenance and repair of DNA, acting on the apoptosis of mutated cells, and is therefore an important tumor suppressor (30). Thus, the loss of mutation-generated p53 function leads to the accumulation of DNA mutations, cell cycle dysregulation and apoptotic induction (31). Therefore, a high proportion of cells with the mutated p53 protein is indicative of greater tumor aggressiveness (6).

Cyclooxygenases (COX-1, COX-2, and COX-3) are enzymes involved in the conversion of arachidonic acid to prostaglandins (32–34). COX-2 is an enzyme induced by inflammatory cytokines and growth factors and it is practically absent in normal tissues. Prostaglandins induce angiogenesis and apoptotic inhibition, contributing to the development of neoplastic processes (32, 35, 36). High expression of COX-1 and COX-2 has already been demonstrated in gliomas and meningiomas in both tumor cells and perilesional macrophages (37), as has the positive correlation between COX-2 and Ki67 expression, suggesting a relation between COX-2 and tumor progression (38). COX-2 expression has also been reported in meningioma stroma macrophages, and one study has questioned whether anti-inflammatory drugs that block COX-2 can inhibit the inflammatory cascade, interfering with tumor growth, particularly in meningiomas with high indices of Ki67 (39).

The *HER2* oncogene is a member of the epidermal growth factor receptor (EGFR) family. This tyrosine kinase receptor encodes the p185 protein and is associated with multiple signals of the transduction pathways involved in cell growth (40). HER2 family receptors are critical for fetal development; however, their presence in adult tissues and their overactivation or dysregulation is related to several tumors, especially breast cancer (41). When active, this receptor binds to dimers, undergoes transphosphorylation and can transduce intracellular signals that may affect cell growth, apoptotic inhibition, migration, invasiveness, and angiogenesis, all of which can lead to tumor progression (41). Its presence is a poor prognostic factor in tumors, particularly breast carcinoma (7, 42, 43). Studies in the literature on this protein in meningiomas are rare (7, 42), and discrepancies between the results are evident (7, 40).

The predominance of meningiomas among females the potential actions of the sex hormones, molecular, inflammatory and growth factors of these tumors led us to analyze their association with clinical and imaging data, and meningioma recurrence.

## Materials and Methods

Ninety-six grade I intracranial meningiomas samples were collected, from patients operated using microsurgical techniques, at the Santa Casa Neurosurgery Service (SCBH), Minas Gerais, Brazil, from December 2009 to June 2015. Epidemiological, clinical, imaging, surgical and follow-up data were recorded using a pre-established protocol. We have considered total resection using Simpson grade I, and II and the partial resection using grade III, IV, and V. The histopathological diagnosis was made by the pathologists of the SCBH Anatomic Pathology Service, using the criteria recommended by the 2007/2016 World Health Organization (WHO) (3, 44). Immunohistochemical expression was performed on 1 mm tissue microarray (TMA) using antibodies specific for HLA-G and HLA-E molecules (MEM-G/9 and MEM-E/02, respectively. ExBio, Praha), cell proliferation indices (MIB-1. Dako, Denmark), PR (PGR363. Dako, Denmark), ER (6F11. Spring), AR (AR441. Dako, Denmark), p53 (DO-7. Dako, Denmark), COX-2 (CX229. Cayman Chemical) and HER2 (SP3. LabVision). Breast carcinoma samples were used as a positive control for Ki67, PR, ER, p53, HER2 and placenta for HLA-G, HLA-E, COX-2, and prostate carcinoma for RA AR. As negative controls, the same cases were used with primary antibody suppression. All laboratory and clinical variables were statistically correlated with each other and with recurrent tumors. The recurrence and regrowth were included in the same group for statistical analysis, which could be considered an analysis bias. The following tests were used: Kolmogorov-Smirnov, Student t, ANOVA, Mann-Whitney, Paired t, Chi-square, Fisher, Stepwise, Pseudo R2, and Hosmer-Lemeshow. The significance level used for the tests was 5%. The survival curve of recurrence/regrowth was calculated by the Kaplan-Meier method and the survival of Simpson grades, tumor size and Ki67 were calculated by the Kaplan-Meier method curves compared by the Log-Rank test. The software used was SPSS for Windows, version 20 (SPSS Inc., Chicago, IL, USA).

This research was approved by the Research Ethics Committee of the SCBH Teaching and Research Institute (IEP), under protocol CEP 664.402, and only the data of patients who accepted and signed a term of free informed consent were included in the analyses.

## Results

Patient mean age was 54.0 ± 14.6 (range 19–80) years old, with a predominance of females (71.9%) and a female:male ratio of 2.6:1. The subtypes of meningiomas identified were: meningothelial (69.9%), transitional (14.6%), fibrous (7.3%), psammomatous (3.1%), angiomatous (1.0%), microcystic (3.1%), secretory (1.0%), lymphoplasmacytic (0%), metaplastic (0%). Preoperative imaging exams detected peritumoral edema in 29.2% of cases, intratumoral calcifications in 10.4% and tumor cysts in 4.2%. Meningiomas were classified according to their largest diameter as follows: small (≤2 cm, 1.0%), medium (>2 and ≤4 cm, 32.3%), and large (>4 cm, 66.7%) (Table 1). For the purposes of statistical analysis, small and medium-sized tumors were considered as one group. The size was not related to recurrence/regrowth (*p* = 0.486) but was significantly correlated with peritumoral edema (*p* = 0.031) and intratumoral calcifications (*p* = 0.018) (Table 2). Just over half (51.0%) of the 96 grade I meningiomas in our series were found at supratentorial locations (Table 3). Total resection was performed in 81.3% of cases and partial resection in 18.7%. Recurrent meningiomas were verified in 14.6% of cases, in the pre- and postoperative periods, at a median postoperative follow-up of 3.6 years.

**Table 1:** Characteristics of meningiomas according to imaging exams.

**Variable**	***n***	**%**
**Tumor size**		
Large (>4 cm)	55	66.7
Medium (>2 and ≤4 cm)	26	32.3
Small (≤2 cm)	1	1.0
Tumor calcification	10	10.4
Presence of cysts	4	4.2
Presence of peritumoral edema	28	29.2

**Table 2:** Correlations between tumor size and calcification, peritumoral edema, and recurrence/regrowth.

**Variables**			**Size**		
	***n***	**Medium**	**Large**	***p*-value**	
Calcification	No	*n*	27	45	**0.018**
		%	100.0	81.8	
	Yes	*n*	0	10	
		%	0.0	18.2	
Peritumoral edema	No	*n*	23	34	**0.031**
		%	85.2	61.8	
	Yes	*n*	4	21	
		%	14.8	38.2	
Recurrence/regrowth	No	*n*	22	48	0.486
		%	81.5	87.3	
	Yes	*n*	5	7	
		%	18.5	12.7	

**Table 3:** Locations of the intracranial meningiomas.

**Location**	***n***	**%**
**Supratentorial**	**49**	**51.0**
Parasagittal/falcine	34	35.4
Convexity	12	12.5
Intraventricular	3	3.1
**Cranial Base**	**33**	**34.4**
Sphenoid wing/orbital	14	14.6
Frontobasal	9	9.4
Sellar/parasellar	9	9.4
Petroclival	1	1.0
**Posterior fossa**	**9**	**9.4**
Pontocerebellar angle	7	7.3
Foramen magnum	2	2.1
**Tentorial** (infra and supra)	**5**	**5.2**

The results for immunostaining are presented in Tables 4, 5. Regarding the markers analyzed, calcifications and peritumoral edema were not significantly related to HLA-E, Ki67, PR, ER, AR (*p* > 0.05). However, recurrent tumors were more frequent in patients with higher Ki67 immunostaining (*p* = 0.014) (Table S1). Regarding the patient age, a significant difference was observed regarding immunostaining of the HLA-E immunoregulatory molecule; HLA-E positive patients were younger than HLA-E negative (HLA-E + 54.01 ± 14.27 and HLA-E – 71.33 ± 7.57; *p* = 0.040). None of the other markers were correlated with age (*p* > 0.05).

**Table 4:** Results of staining for Ki67.

**Mean/SD (Amplitude)**	**Median (P25 and P75)**
**Positivity (%)**	
2.61 ± 2.29% (0–16%)	2.10% (1.40 and 3.40%)

*Ki67, cell proliferation index by MIB-1/Ki67 monoclonal antibody*.

**Table 5:** Results of staining for other immunomarkers.

**Markers**	**Positivity**	
	**n**	**%**
COX-2	93	100
p53	88	92.6
PR	57	62
HLA-E	87	95.6
HLA-G	96	100
AR	6	6.5
ER	2	2.1
HER2	0	0

*COX-2, cyclooxygenase 2; p53, nuclear phosphoprotein; PR, progesterone receptor; HLA-E, human leukocyte antigen E; HLA-G, human leukocyte antigen G; AR, androgen receptor; ER, estrogen receptor; HER2, human epidermal growth factor receptor 2*.

According to Abry et al. (45), in grade I meningiomas, the cut-off point for Ki67 immunostaining was 3% of stained cells. Following this cut-off value, Ki67 also showed significant differences when meningiomas were analyzed by subtypes. A higher proportion of Ki67-positive meningiomas were transitional (32.1%), while fibrous (11.7%) and meningothelial (80.0%) meningiomas were more frequently Ki67-negative (Table 6). Considering the 3% cut-off for Ki67, it was not observed any significant difference for other variables. Variables with *p*-values <0.20 in Table 5 were inserted in the multiple logistic regression model (Table 7).

**Table 6:** Ki67 analysis considering a cut-off point of 3% (45), in relation to epidemiological variables, immunomarkers, imaging, histopathology, and clinical evolution.

**Variable**			**Ki67**		**Total**	***p*-value**
			**≤3%**	**>3%**		
Sex	Female	*n*	48	21	69	0.938
	Male	%	71.6	72.4	71.9	
	Male	*n*	19	8	27	
		%	28.4	27.6	28.1	
Total		*n*	67	29	96	
ER %cell	–	*n*	65	27	92	0.509
		%	98.5	96.4	97.9	
	+	*n*	1	1	2	
		%	1.5	3.6	2.1	
Total		*n*	66	28	94	
PR %cell	–	*n*	22	13	35	0.273
		%	34.4	46.4	38.0	
	+	*n*	42	15	57	
		%	65.6	53.6	62.0	
Total		*n*	64	28	92	
p53	–	*n*	4	3	7	0.433
		%	6.1	10.3	7.4	
	+	*n*	62	26	88	
		%	93.9	89.7	92.6	
Total		*n*	66	29	95	
AR %cell	–	*n*	62	25	87	0.361
		%	95.4	89.3	93.5	
	+	*n*	3	3	6	
		%	4.6	10.7	6.5	
Total		*n*	65	28	93	
**Size**	Medium	*n*	17	10	27	0.367
		%	29.8	40.0	32.9	
	Large	*n*	40	15	55	
		%	70.2	60.0	67.1	
Total		*n*	57	25	82	
**Calcification**	No	*n*	62	24	86	0.163
		%	92.5	82.8	89.6	
	Yes	*n*	5	5	10	
		%	7.5	17.2	10.4	
Total		*n*	67	29	96	
**Peritumoral edema**	No	*n*	49	19	68	0.451
		%	73.1	65.5	70.8	
	Yes	*n*	18	10	28	
		%	26.9	34.5	29.2	
Total		*n*	67	29	96	
**Recurrence/** **regrowth**	No	*n*	60	22	82	0.114
		%	89.6	75.9	85.4	
	Yes	*n*	7	7	14	
		%	10.4	24.1	14.6	
Total		*n*	67	29	96	
HLA-E %cell	–	*n*	3	0	3	HLA-E %cell
		%	4.8	0.0	3.3	
	+	*n*	59	28	87	
		%	95.2	100.0	96.7	
Total		*n*	62	28	90	Total
**Follow-up**	Alive	*n*	52	23	75	0.853
		%	77.6	79.3	78.1	
	Dead	*n*	15	6	21	
		%	22.4	20.7	21.9	
Total		*n*	67	29	96	
**Subtypes of meningiomas**	Fibrous	*n*	7	0	7	**0.005**
		%	11.7	0.0	8.0	
	Meningothelial	*n*	48	19	67	
		%	80.0	67.9	76.1	
	Transitional	*n*	5	9	14	

*Ki67, cell proliferation index by MIB-1/Ki67 monoclonal antibody; ER, estrogen receptor; PR, progesterone receptor; p53, nuclear phosphoprotein; AR, androgen receptor; HLA-E, human leukocyte antigen E. Chi-square test*.

**Table 7:** Multiple logistic regression of recurrence/regrowth, calcification and histology in relation to Ki67.

**Variables inserted in**	**B**	**S.E**.	**Wald**	**df**	***p*-value**	**OR**	**95%CI for OR**	
**the logistic regression**								
Recurrence/regrowth	1.175	0.636	3.412	1	0.065	3.238	0.931	11.264
Calcification	1.090	0.750	2.110	1	0.146	2.973	0.684	12.930
Grade I subtypes			1.921	2	0.383			
Meningothelial	0.889	0.641	1.921	1	0.166	2.432	0.692	8.551
Transitional	−20.306	16,148.080	0.000	1	0.999	0.000	0.000	
Constant	−1.149	0.315	13.294	1	0.000	0.317		

*Ki67, cell proliferation index by monoclonal antibody MIB-1/Ki67; CI, confidence interval*.

*Correct classification percentage = 72.7%; Pseudo R2 = 0.180; Hosmer-Lemeshow Test = 0.912*.

Evaluation of the parameters regarding the adequacy of the model (% of correct classification, pseudo R2 and the Hosmer-Lemeshow test) showed there was no violation. However, the explanatory percentage of the model (R2) is very low (18%), which indicates that the variables together do not clearly explain the results for Ki67. No joint effect of the variables was observed (Table 7).

**Figure 1:**
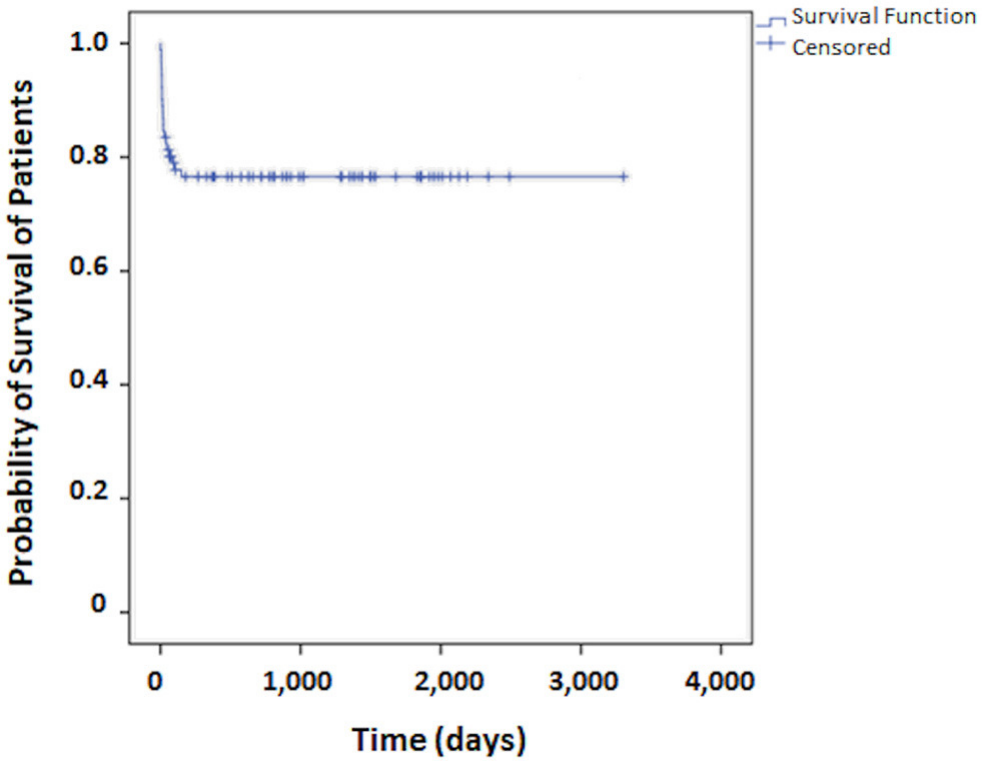
Survival curve for patients who undergo surgical treatment of meningioma.

**Figure 2:**
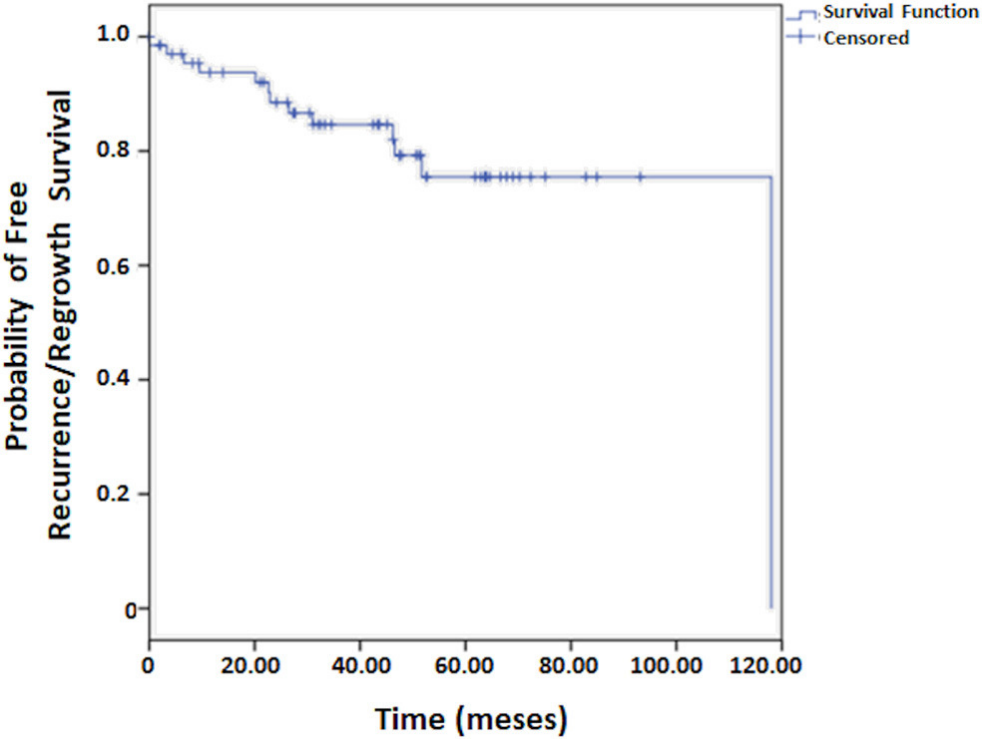
Free recurrence/regrowth survival for patients who undergo surgical treatment of meningioma.

**Figure 3:**
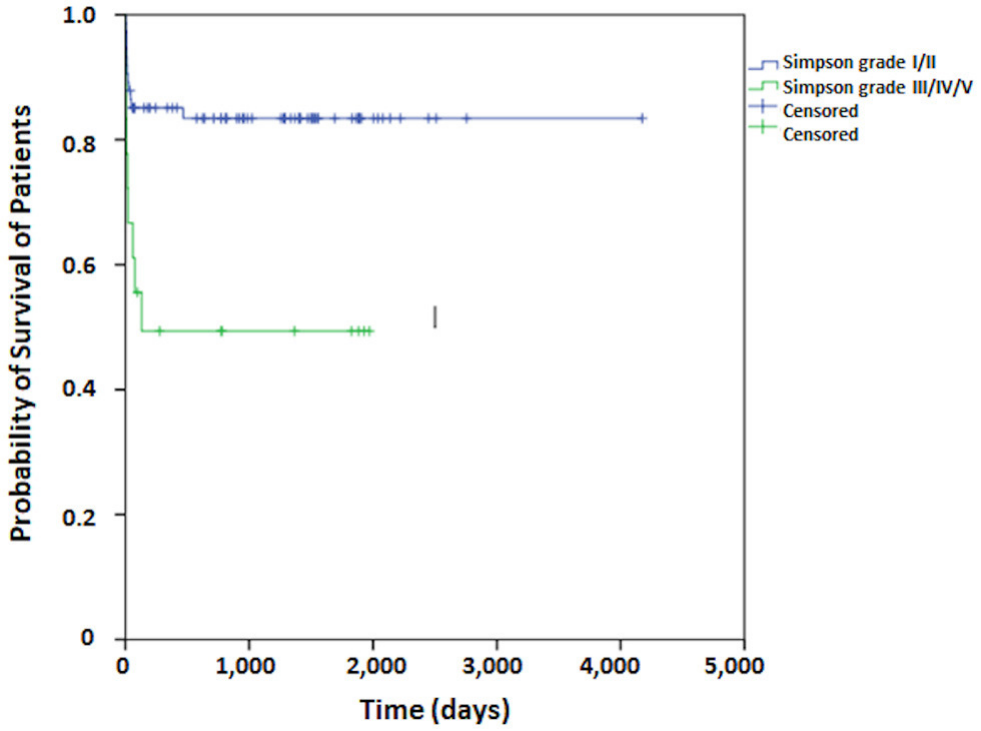
The survival curve by the Kaplan-Meier method compared by the Log-Rank test concerning to Simpson's tumor resection degrees for patients who undergo surgical treatment of meningioma.

**Figure 4:**
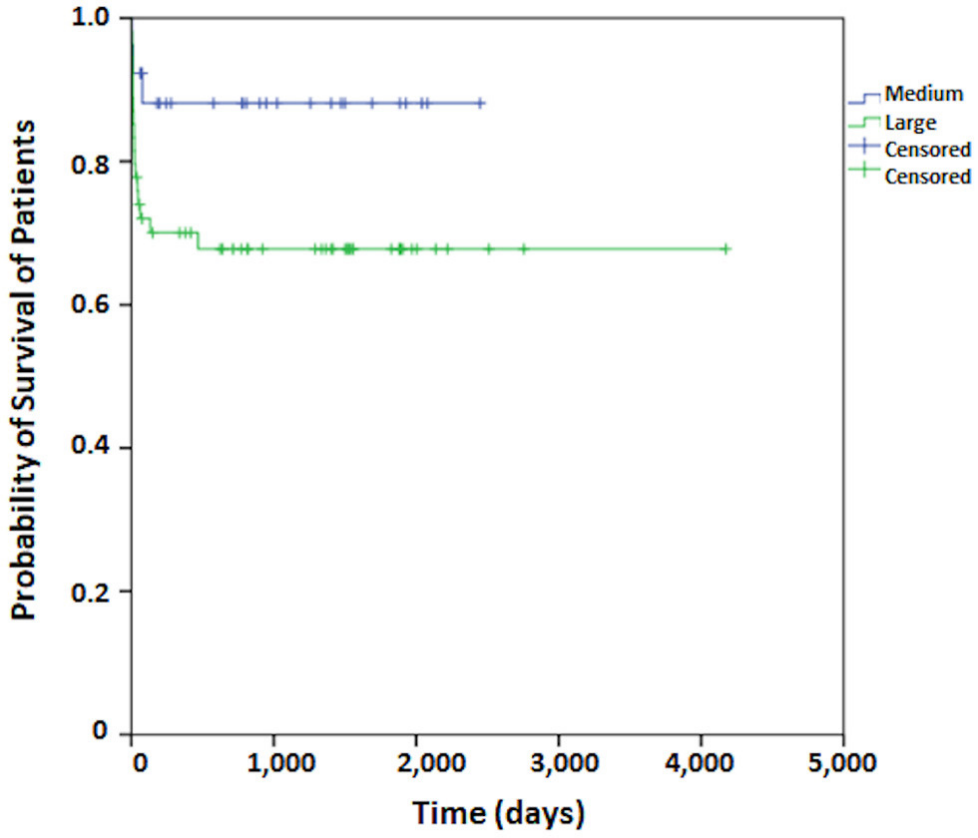
The survival curve by the Kaplan-Meier method compared by the Log-Rank test concerning to tumor size for patients who undergo surgical treatment of meningioma.

**Figure 5:**
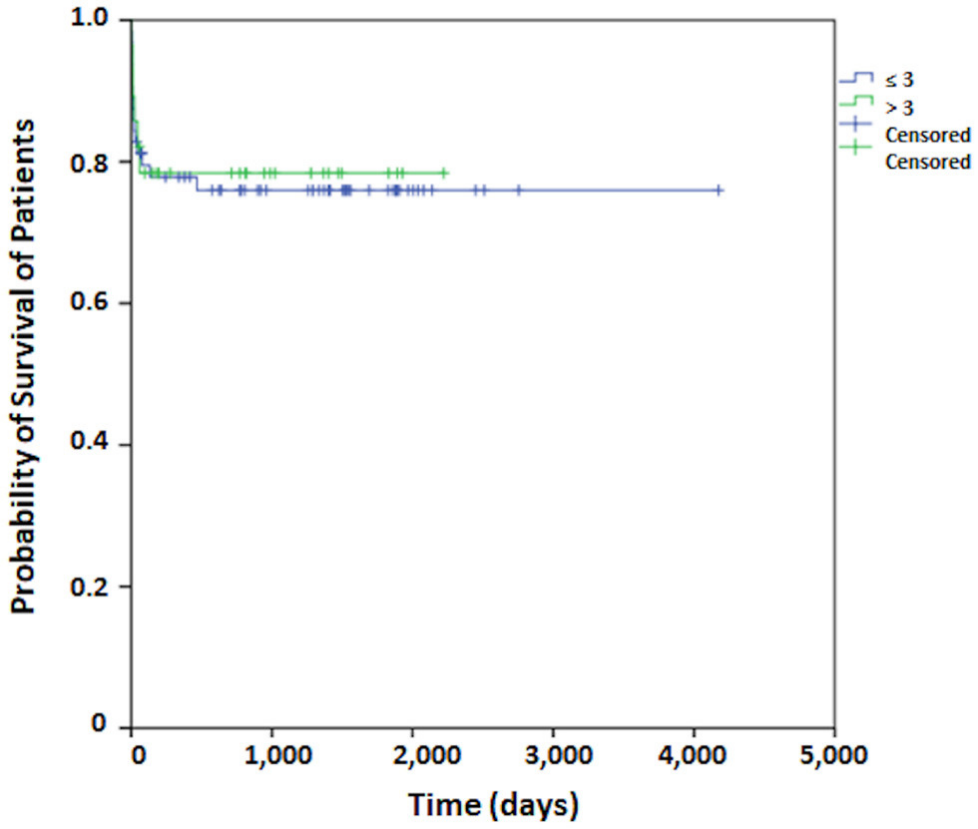
The survival curve by the Kaplan-Meier method compared by the Log-Rank test concerning to cut-off for Ki67 for patients who undergo surgical treatment of meningioma.

Regarding patient survival, at 30 days it was 83.5%, at 60 days, 80.2%, at 90 days, 79.0%, and at 120 days it was 77.8%. From this point onward, survival remained stable. The median period for patient follow-up was 3.6 years (Figure 1). The Kaplan-Meier curve for free recurrence/regrowth survival was obtained for thirteen patients and, at 12 months it was 93.7%, at 24 months, 88.5%, at 36 months, 84.6%, at 48 months it was 79.3%, and at 60 months, it was 75.5% (Figure 2; one patient was not included in these analyses due to insufficient data). The survival curve was analyzed by the Kaplan-Meier method and compared by the Log-Rank test concerning to Simpson's tumor resection degrees, at 2 months it was in grades I/II 85.1% (IC 95%:75.9–94.3%) and grades III/IV/V 66.7% (IC 95%: 44.7–88.7%), at 18 months it was in grades I/II 83.4% (IC 95%: 75.9–94.3%) and grades III/IV/V 49.4% (IC 95%: 27.4–71.4%); *p* = 0.001 (Figure 3). The survival curve was analyzed by the Kaplan-Meier method and compared by the Log-Rank test concerning to tumor size, at 2 months it was in median size 92.3% (IC 95%:82.0–100%) and in large size 72.0% (IC 95%: 60.0–84.0%), at 18 months it was in median size 88.1% (IC 95%: 76.0–100.0%) and in large size 67.8% (IC 95%: 55.8–78.8%); *p* = 0.064 (Figure 4). The survival curve was analyzed by the Kaplan-Meier method and compared by the Log-Rank test concerning to cut-off for Ki67, at 2 months it was in ≤3% 81.2% (IC 95%: 72.2–90.2%) and in >3% 78.4% (IC 95%: 64.4–92.4%), at 18 months it was in ≤3% 76.0% (IC 95%: 66.0–86.0%) and in >3% 74.4% (IC 95%: 64.4–92.4%); *p* = 0.841 (Figure 5).

## Discussion

### Clinical, Imaging, and Surgical Data

Our analyzes confirmed that the larger the grade I meningioma, the higher the number of calcifications and the more severe the peritumoral edema. Regarding calcifications and tumor size, our results disagree with the findings reported by Nakasu et al. (46). Regarding peritumoral edema and tumor size, our results corroborate those reported in the literature (47, 48). Recurrence/regrowth was not associated with tumor size, but the percentage obtained herein was consistent with that described by other authors, who reported recurrence rates in grade I meningiomas of up to 20%, particularly tumors showing higher positivity for Ki67 (5, 6, 11, 45, 49–53). In our study the free recurrence/regrowth survival at 12 months was 93.7% and at 60 months, it was 75.5%. Considering ours results on recurrence/regrowth-free survival curves, Simpson's degree of resection, tumor size and Ki67, the only one that shows a statistically significant value is the degree of resection (*p* = 0.001). Therefore, an increase on the disease-free survival is associated with larger extension of resection (Simpson I/II). The location of meningiomas in our series is similar to that of some series (1, 51, 54) while it diverges from others (27, 55). Bhat et al. determined that 89.3% of their series of 729 meningiomas were grade I (1). Among their cases, 50.4% were supratentorial, 33.7% were located at the base of the skull, 9.3% in the posterior fossa, and 6.6% in the tentorial region (1).

### Immunomarkers

#### HLA-G and HLA-E Molecules

In this study, no significant correlations were observed between staining for HLA-E, Ki67 and PR immunomarkers and the presence of calcifications, peritumoral edema and recurrence/regrowth. The only relation verified was with patient age, where HLA-E+ patients were significantly younger than HLA-E– patients, which seems to suggest that the levels of this molecule decrease with age. If this were true, however, we would expect to see less immunosuppressive action for HLA-E, with a decrease in the incidence of meningiomas in older adults. This directly contradicts the results reported by Ostrom et al. (2), who demonstrated an exponential increase in meningioma incidence from the age of 65. It may be more useful to focus on immunosuppression, and the possible involvement of HLA-G molecules in this event, because among our samples, HLA-G showed 100% immunostaining in all cases of meningiomas, and this could explain the presence of this tumor type in patients of all ages. HLA-G expression has been reported to be very heterogeneous in different types of tumors (19, 20, 56) and that even a low cellular expression of this molecule (around 10%) is sufficient to induce immunotolerance (56). Perhaps similar effects apply to HLA-E.

To date, no other studies evaluating the tissue expression of HLA-G in meningiomas have been published. The only existing work was conducted by our group on plasma from patients with malignant gliomas and other benign intracranial tumors. Comparisons between these groups showed that HLA-G plasma expression in meningiomas was significantly lower than in multiform glioblastomas, admittedly the most aggressive of adult primary brain tumors (24). In our research, all the meningioma samples presented 100% HLA-G positivity, thus it was not possible to perform statistical analyses with the other variables. We risk the hypothesis that the high HLA-G positivity in our samples could be contributing to the high recurrence/regrowth rates (14.6%) in grade I meningiomas by inhibiting immune surveillance. However, these data require more rigorous confirmation, since these analyses were based on TMA immunostaining. Perhaps this technique is not the most suitable for evaluating HLA-G, as reported by Arnli et al. (57) for HER2, where they observed differences in the intensity of HER2 immunostaining in different sections of the meningioma, suggesting that the TMA technique can lead to misinterpretation. On the other hand, Lusis et al. (58) reported that the analysis of positivity for PR immunomarkers, epithelial membrane antigen (EMA), cathepsin D, E-cadherin, platelet-derived growth factor beta receptor (PDGF), epithelial growth factor receptor (EGFR), and vascular endothelial growth factor (VEGF) with the use of immunohistochemistry by TMA showed very similar efficiency with all sections of the lesion. This theme requires further controlled studies, because each marker may present differently.

#### Cell Proliferation Index: Ki67

Ki67 correlates with the mitotic index and has been used in breast cancer as a prognostic marker and the responses to endocrinological treatment and chemotherapy (26). However, in other tumor types, including meningiomas, the definition of a cut-off point for Ki67 remains undefined (26). In a meta-analysis evaluating Ki67 positivity in grade I meningiomas, the authors achieved average immunostaining of around 3% (range 1–16%), and around 4% (range 0–35%) in recurrent tumors (45). In other studies, meningiomas presenting Ki67 ≥ 3% also showed a greater tendency for recurrence (5, 10, 52, 55, 59–64). In contrast, some studies have not confirmed this trend based only on Ki67 (13, 65, 66). In our analyzes, no significant differences were observed in Ki67 for grade I meningioma subtypes, sex, tumor size, intratumoral calcifications, peritumoral edema, or surgery-related deaths. Similar findings were observed for the other immunomarkers we studied. Regarding sex, data from the literature is controversial, with some authors observing no association between Ki67 and sex (52, 66, 67), while others reported a positive relation with males (45) or females (68).

Considering tumor size and peritumoral edema, in contrast to our results, Pavelin et al. (31), reported significantly positive relations between both Ki67 and p53 and tumor size, concluding that they probably influence meningioma development and growth (31). However, it is important to consider that grade I meningiomas present slow growth, consequently resulting in signs and symptoms in older adults, and generally present low Ki67 rates. Regarding peritumoral edema, no relation was observed with Ki67 (*p* = 0.120) in this study, in disagreement with the results of other authors who reported an association for this marker with peritumoral edema in meningiomas (25, 47, 67, 69, 70). Perhaps Ki67 contributes to increased vascular neoformation favoring the formation of peritumoral vasogenic edema (66).

In this study, Ki67 was only related to recurrence/regrowth, suggesting that it could influence the growth of grade I meningiomas. This result agrees with the literature, which shows that the mean positivity of Ki67 in grade I meningiomas ranges from 1 to 4%, and our result is within this range (9, 28, 51, 71–73) (see the Summary, Table S2). Considering the 3% Ki67 cut-off point in grade I meningiomas, suggested by Abry et al. (45), we observed a significant difference between the degree of positivity according to the type of meningioma: transitional meningiomas were more positive (32.1%) than meningothelial (67.9%) and fibrous meningiomas (0.0%) (*p* = 0.005) (Table 6). However, this result is not corroborated by findings described in the literature, which show no differences in positivity for Ki67 in subgroups of the same grade of meningioma (27, 66). Moreover, in contrast to that observed herein, greater immunostaining of Ki67 was observed in meningothelial meningiomas (9, 31). This may be due to methodological differences in the classification of these subtypes of meningioma.

Since the biological behavior of grade I meningiomas indicates they are prone to recurrence, they cannot be considered benign entities, despite their histopathological classification. The concept of radical or total resection is subjective, and may influence the differences in the percentage of recurrence between studies (66). Even grade I tumors can invade the dura and adjacent bones and tumor cells not visualized during surgery may be missed, contributing to recurrence (55).

In a study of 162 meningiomas submitted to total resection, excluding those with convexity, and follow-up of 2–5 years (mean of 3 years), recurrence occurred in 32.7% of meningiomas, 25.4 % among grade I tumors (10), a result that is higher than that observed in our research. According to Tao et al. (10), the median time to recurrence was ~3 years. In this study, the median postoperative follow-up was 3.6 years, i.e., within the period that presents a higher number of recurrences. According to Tao et al. (10), the higher the histological grade, Ki67, ER and AR, the higher the number of recurrences, and the finding was significant (*p* < 0.05). They also reported that grade I meningiomas have a recurrence risk of 7–20% after surgical resection, and this was significantly higher in meningiomas with high Ki67 (10), a finding also observed in our study. A higher number of recurrences was also observed in tumors larger than 4 cm, in irregular tumors and with longer follow-up (10). In contrast, we observed no significant relation between large tumors and recurrence. In the study by Tao et al. (10), the most recurrent meningiomas were those where postoperative tumor remnants were recorded, such as tumors located near the superior sagittal sinus, falx cerebelli and skull base, since total resection in these areas is impossible and only coagulation of the tumor implantation on the dura mater or infiltrated venous sinuses is performed (Simpson II) (74).

Histology alone is not always predictive of the biological behavior of meningiomas. The cell proliferation index (Ki67) has been shown to be of great value in identifying the most aggressive meningiomas. In a meta-analysis of 53 articles, totaling 6,498 meningiomas, a positive correlation between Ki67 and the grade of meningioma was verified. The mean of Ki67 indices for grade I was 3% (range 1–16%), grade II was 8% (range 2–20%), and grade III was 17% (range 7–32%). Primary meningiomas had a mean Ki67 of 2% (range 0–20%) and recurrent of 4% (range 0–35%). Meningiomas with a Ki67 of >4% were indicative of higher recurrence rates. However, some benign meningiomas (grade I) may present a high Ki67 and should be followed closely due to the possibility of more aggressive behavior. In our series, the mean Ki67 was 2.61 ± 2.29% (range 0–16%), while in primary tumors it was 2.45 ± 2.27% and recurrent tumors it was 3.52 ± 2.25%, in agreement with the study by Abry et al. (45). Thus, Ki67, together with the histopathological features of malignancy, may serve as a potential indicator of the risk of meningioma recurrence (45).

Several immunomarkers have been suggested as predictive of recurrence. Despite this, only Ki67 is routinely used in clinical practice. In a study on 59 grade I meningiomas (38 primary, 21 recurrent), with follow-up of 6–16 years, 22 immunomarkers were analyzed, including Ki67, PR, ER, AR and p53 (13). No significant value for these immunomarkers was determined in relation to recurrences. In general, Ki67 showed a range from 0 to 10%, with primary tumors presenting 3.34 ± 0.4% and recurrent tumors 3.9 ± 0.5%. These results were not significant, in contrast to our study, and according to Kärjä et al. (13), only the degree of tumor resection and a long follow-up period were significantly related to the risk of recurrence.

Given these findings, prognosis in meningiomas cannot be determined solely based on the histology, the degree of resection, location or Ki67 (75). Thus, further standardized studies are required to more clearly define this controversial theme.

#### Progesterone, Estrogen, and Androgen Receptors

Our results for the percentage of PR expression were similar to those of other authors (51, 76–78) and in disagreement with others (73, 79–81). There was broad variation in grade I meningiomas from 22.9 to 100%, with high expression in most studies (Table 8). Discrepancies in PR immunoreactivity may be due to different sensitivities among the antibodies used.

**Table 8:** Series of grade I meningiomas and respective percentages of PR expression.

**Authors, year,** **country**	**No. of cases of** **grade I meningiomas**	**Percentage of PR** **immunoreactivity ± SD**	**Relation between PR+ and** **PR- and recurrence: (*p*-value)**
Current paper	96	62.0	*p* = 0.845
Fewings et al., 2000, England (79)	60	48.0	46 with long FU more recurrent tumors in PR- (*p* = 0.013)
Das et al., 2002, Thailand (76)	90	67.0	
Konstantinidou et al., 2003, Greece (82)	38	75.5	More recurrent tumors in PR-
Wolfsberger et al., 2004, Austria/Germany (81)	51	22.9 ± 3.7	
Roser et al., 2005, Germany (77)	385	<70 years: 56.1 ≥70 years: 58.4 *p* > 0.05	
Lusis et al., 2005, USA (58)	5	100.0	
Korhonen et al., 2006, Finland (11)	407 primary tumors	88.8	*p* = 0.43
Omulecka et al., 2006, Poland (62)	46	Meningothelial 100.0 Transitional 95.2 Fibrous 42.2	
Maiuri et al., 2007, Italy (63)	57	Recurrent 18.5 Primary 30.0	more recurrent tumors in PR- (*p* = 0.0001)
Taghipour et al., 2007, Iran (78)	51	68.6	
Takei et al., 2008, USA (9)	46	92.1	
Kandemir et al., 2010, Turkey (71)	53	50.9	
Kärjä et al., 2010, Finland/Sweden (13)	59	Recurrent 47.6 ± 9.2 Primary 48.9 ± 6.8	*p* = 0.331
Shayanfar et al., 2010, Iran (80)	63	96.8	
Abdelzaher et al., 2011, Egypt (51)	60	70.0	*p* > 0.05
Iplikcioglu et al., 2014, Turkey (14)	26	73.0	*p* = 0.69
Mukhopadhyay et al., 2017, India (73)	82	96.34	

*PR, progesterone receptor; PR+, positive immunoreactivity for PR; PR-, negative immunoreactivity for PR; SD, standard deviation*.

Most studies reported an inverse association between PR and Ki67 immunomarker positivity, that is, in PR+ tumors Ki67 positivity is lower, indicating less proliferative activity and a less aggressive tumor. In RP- tumors, Ki67 was generally higher, presenting the most aggressive behavior (9, 52, 58, 62, 66, 80, 82). However, in our analyses, we observed no significant differences between these markers (*p* = 0.790), a result agrees with other studies (11, 77). According to Konstantinidou et al. (82), the presence of PR– is a predictor of early recurrence and PR+ is associated with a favorable prognosis in breast cancer, similar to that described for meningiomas.

In our study, no significant relations were observed between PR expression and sex, age, intratumoral calcifications, tumor size, peritumoral edema, HLA-E, Ki67, p53, or between primary and recurrent meningiomas.

Regarding patient sex, several authors also observed no association with RP expression (11, 62, 66, 71, 77), but others reported positivity for PR in females (14, 25, 52, 68, 78, 80–86). One study showed the expression of PR+ in 91% of males and 81% of females with meningiomas (87), and other work showed a predominance of PR expression in males under 50 years old (81). It is possible that these discrepancies in the results are due to methodological and population differences. Regarding age, we observed no significant differences between PR+ and PR– (*p* = 0.747), in agreement with some studies (11, 77). In disagreement with our results, another study on 50 meningiomas (84% grade I, 10% grade II and 6% grade III) observed a significant relation between age and PR expression (*p* = 0.021), but not in relation to Ki67 (52). In our study, no relation between PR expression and peritumoral edema (*p* = 0.123) was verified. In contrast, another study showed that tumor size and the presence of peritumoral edema were statistically greater in PR– meningiomas (25). We observed no significant differences between primary and recurrent grade I meningiomas in relation to PR+ and RP– (*p* = 0.845), in agreement with other works (9, 11, 13, 14, 51, 77). However, some studies have reported a higher number of recurrences in PR- in meningiomas (63, 79, 82). Thus, further studies are required to determine whether PR is associated with peritumoral edema and recurrence in meningiomas (Table 8).

Regarding estrogen receptors, we verified low mean expression for this marker in our samples, which was consistent with the findings of other authors (51, 88). Considering ER expression only in grade I meningiomas in the literature, the mean ranges from 0 to 41.6% in four studies (11, 13, 51, 71), two of which reported values well above our result (11, 13). However, of the 15 studies cited, including all grades of meningioma, five of these were unable to determine ER expression, and three others showed low ER expression. Therefore, low ER or the absence of ER expression corresponded to the majority (8/15) of these studies (Table 9), and our results further corroborate these findings.

**Table 9:** Series of meningiomas and respective percentages of ER expression.

**Authors, year, country**	**ER % ± SD**	**Observations**
Current paper	2.1	96 grade I
Korhonen et al., 2006, Finland (11)	41.6	407 grade I primary tumors. Weak immunoreactivity.
Kandemir et al., 2010, Turkey (71)	0	53 grade I
Kärjä et al., 2010, Finland/Sweden (13)	Primary: 40.0 ± 2.5 Recurrent: 33.3 ± 3.3	59 grade I (*p* = 0.105)
Abdelzaher et al., 2011, Egypt (51)	3.33	60 grade I
Blankenstein et al., 2000, Holland (89)	13.0	396 grades I, II and III. Predominance of ER/PR+
Konstantinidou et al., 2003, Grécia (82)	35.4	49 grades I and II
Barbosa-Coutinho, Hilbig, 2006, Brazil (54)	0	Grades I, II, and III
Custer et al., 2006, USA (88)	1.0	140 grades I, II, and III
Omulecka et al., 2006, Poland (62)	48.0	46 grade I and 18 grade II (*n* = 64) No relation with grade, sex, Ki67 or PR
Taghipour et al., 2007, Iran (78)	0	50 grades I, II, and III
Takei et al., 2008, USA (9)	10.4	46 grade I and 11 grade II (*n* = 57) No relation with grade, Ki67 or PR
Hirota et al., 2009, Japan (90)	0	82 grades I, II, and III
Leães et al., 2010, Brazil (86)	24.6	126 grades I, II, and III
Tao et al., 2012, China (10)	60.5	162 grades I, II, and III. Weak expression for ER. No relation with recurrence.
Iplikcioglu et al., 2014, Turkey (14)	0	26 grade I and 24 grades II and III

*ER, estrogen receptor; SD, standard deviation*.

We determined no significant relation between age and ER (*p* = 0.884), nor between primary and recurrent meningiomas with this marker (*p* = 0.626), in agreement with the results of another study (11). In contrast, one study showed ER+ in patients over 60 years old (82). ER expression was also not related to the patient sex, a fact observed in other studies (11, 62); however, Konstantinidou et al. (82) reported higher ER expression in females. We observed no significant relation between ER and Ki67, similar to a study by Omulecka et al. (62), but in disagreement with another study, which showed higher Ki67 in grade I meningiomas with ER+ than with ER– (*p* = 0.038) (11). Tao et al. (10) reported a higher number of recurrences in meningiomas expressing ER and AR, while Liu et al. (91) affirmed that only low PR expression was related to recurrence, when evaluating sex hormone receptors in meningiomas. Despite these findings, low ER expression in the great number of the studies and the absence of a relation with recurrences suggest that ER does not influence the pathogenesis and growth of meningiomas.

In our research, low expression of AR (6.5%) was observed, in contrast to the literature, in which the values for grade I meningiomas range from 31.0 to 40.3% (11, 13, 92), and in all grades from 18.3 to 67% (82, 86, 93) (Table 10). These variations may be due to differences in the immunomarker and/or population sensitivities. We observed no significant correlations between AR expression and age (*p* = 0.677), Ki67 (*p* = 0.097), or primary and recurrent meningiomas (*p* = 1.000). In line with our results on age and recurrence, an analysis of 407 primary meningiomas and 37 grade I recurrences reported no significant differences in AR expression between them, nor in relation to patient sex (11). In another study, weak or positive AR expression was observed in 38.8% of the 162 meningioma specimens (grades I, II and III) studied using immunohistochemistry on TMA sections. A positive relation between recurrence and AR+ expression was evidenced in monofactorial analysis, but this was not confirmed in the multifactorial regression analysis (10), in agreement with our study. In disagreement with our work, immunohistochemical analysis of 39 meningiomas of all grades showed AR was expressed in varying intensities in 67% of cases, with a predominance for females (86%) compared with males (44%) and the finding was significant. This female predominance has been reported in previous studies (94, 95). Other studies have reported a positive relation between high Ki67 expression and increased AR expression (82, 92). Immunostaining of AR was observed in proliferative endothelial cells of tumor microvasculature, together with nuclear labeling, leading to the conclusion that AR was related to Ki67 and tumor grade, and probably participates in the growth processes and neoangiogenesis of meningiomas (92). On the other hand, low AR expression and the absence of a relation with recurrence in most studies suggest that AR does not influence on the pathogenesis and growth of grade I meningiomas.

**Table 10:** Series of meningiomas and respective percentages of AR expression.

**Authors, year, country**	**AR %**	**Observations**
Current paper	6.5	96 grade I
Carrol et al., 1995, USA (93)	67.0	39 grades I, II and III
Chen and Chen, 2001, China (92)	31.0	19 grade I
Konstantinidou et al., 2003, Greece (82)	28.6	39 grades I, II, and III
Korhonen et al., 2006, Finland (11)	40.3	47 grade I primary tumors. No relation to recurrent tumors
Kärjä et al., 2010, Finland /Sweden (13)		59 grade I. Primary, 32.3 ± 2.5%; recurrent, 32.4 ± 2.4%; *p* = 0.881
Leães et al., 2010, Brazil (86)	18.0	126 grades I, II, and III. No differences observed between subtypes

*AR, androgen receptor*.

The high incidence of meningiomas in females cannot be explained solely by the differences in the expression of sex hormone receptors. Sex hormones can influence meningioma growth through other pathways independent of sex hormone receptors (11, 13, 96). We observed no significant relation between grade I meningioma recurrence/regrowth and any of the sex hormone receptors. Tao et al. (10) and Iplikcioglu et al. (14) concluded that PR expression was not related to the biological behavior of meningiomas. In contrast, Pravdenkova et al. (8) demonstrated that PR+ meningiomas presented more favorable outcomes than PR– and ER– tumors, while those that were ER+ presented worse outcomes. Given these findings, hormone receptors should always be studied to assist in the prognosis of meningiomas, especially in females, and histological grade should also be taken into account (8). The controversy in the literature indicates more studies are required to determine whether sex steroid hormone receptors influence the pathogenesis and growth of meningiomas.

#### p53

The *p53* showed high expression in this study, well above that obtained by other authors (6, 13, 76, 97–103). Pavelin et al. (31) described p53 expression ranging from 10 to 88% (Table 11). We also observed no significant relation between p53 immunoreactivity and PR+ (94.7%) or PR– (88.6%) (*p* = 0.421). However, several studies have shown that hormone-dependent tumors, such as endometrial and breast tumors, show an inverse relation between negative or low PR expression and high expression of the p53 tumor suppressor, indicating a worse prognosis (105–108). The role of p53 in meningiomas is controversial and the tendency in the literature indicates that p53 inactivation is associated with meningioma progression (31, 76, 102, 109).

**Table 11:** Series of meningiomas and respective percentages of p53 expression.

**Authors, year, country**	**p53 %**	**Observations**
Current paper	92.6	96 grade I. No relation with PR
Das et al., 2000, Thailand (99)	5.0 grade I	15% in grades I and II
Das et al., 2002, Thailand (76)	14.0	MDM2 expression in 46. Combination of p53 and MDM2 correlated with PR+
Lanzafame et al., 2000, Italy (100)	57.0	69 grades I, II, and III. Greater in grades II and III
Amatya et al., 2001, Japan (101)	19.8	146 cases. Grade I < grade II < grade III
Yang et al., 2008, South Korea (102)	10.0 grade I	25% in grade II and 79% in grade III
Kärjä et al., 2010, Finland/Sweden (13)		59 grade I. Primary, 3.2 ± 1.5%; recurrent, 6.5 ± 3.6%; *p* = 0.889
Abdelzaher et al., 2011, Egypt (51)	6.63 grade I	60 grade I
Pavelin et al., 2013, Croatia (31)		170 cases. No relation with tumor grade. 10–88% of p53 in the literature
Trott et al., 2015, Brazil (6)	46.8 grade I	141 grades I; 13 grades II (84.6%); 3 grades III (33.3%); *p* > 0.05
Fukami et al., 2016, Japan/Germany (104)	47.5	52 grade I, 3 grade II and 4 grade III

*PR, progesterone receptor; MDM2, mouse double minute 2 homolog*.

In our study, we observed no relation between Ki67 and p53, in contrast with some studies. Their research affirms that grade I meningiomas with high expression of p53 and Ki67 tend to recur (31, 109).

One study reported that p53 expression was associated with higher histological grades (II and III) and that the mutated p53 protein showed higher expression in older patients compared with youths (103). This finding is similar to the results of a study by our group, which observed immunostaining for mutated p53 in 90.6% of grade I meningiomas. In that study, immunostaining was strong in 84.4% and weak in 15.6%, particularly in females and in older adults, who are more likely to present higher expression of the mutated protein (110).

#### COX-2

Herein, COX-2 was expressed in 100% of valid cases, thus statistical analysis in relation to other immunomarkers was precluded. In another study by our group, strong labeling of COX-2 immunoreactivity was observed in 86% of meningiomas and weak in 13.6%, predominantly in females (110). According to the literature, meningiomas generally present high COX-2 expression, especially in grades II and III (32, 39, 111). However, others studies have not shown any relation between COX-2 expression and different grades of meningioma (35, 72, 112) (Table 12). The possibility of COX-2 blockade was raised with the use of non-steroidal anti-inflammatory drugs in aggressive meningiomas, including grade I, which present more severe peritumoral edemas (39, 111).

**Table 12:** Series of meningiomas and respective percentages of COX-2 expression.

**Authors, year, country**	**COX-2 %**	**Observations**
Current paper	100.0	96 grade I
Ragel et al., 2005, USA (112)	87.0 grade I	128 grade I. 86% in 7 grade II, in cell cultures
Buccoliero et al., 2007, Italy (35)	83.0	23 meningiomas of all grades
Pistolesi et al., 2007, Italy (111)	82.14	46 grade I, 8 grade II and 2 grade III. Higher COX-2 in higher grades
Lee et al., 2014, South Korea (72)	12.2 grade I	49 grade I, 33 grade II (COX-2, 12.1%), 6 grade III (COX-2, 16.6%). Total of 88 cases.
Kato et al., 2014, Japan (39)	59.1 grade I	44 grade I.

*COX-2, cyclooxygenase 2*.

The high expression of COX-2 in meningiomas indicates that it may form part of the pathogenesis of this tumor type (36). Since COX-2 is found in the leptomeninges endothelium and in the arachnoid cells not affected by meningioma (36), further studies are required to determine whether COX-2 participates in the pathogenesis and development of meningiomas.

#### HER2

The HER2 expression in meningiomas of all grades ranges from 2.0 to 100.0% (40); however, there are few studies on HER2 in meningiomas in the literature (7, 42), and discrepancies between results (7, 40).

It has been reported that HER2 is most commonly found in meningiomas with epidermal differentiation, such as meningothelial and secretory tumors (113). In grade I meningiomas, the high immunoreactivity of this molecule has been observed in 22.6% of 53 cases, mainly in meningothelial tumors (71), but this positivity in meningothelial tumors was not confirmed in another study (114). In our samples, there was no immunostaining for HER2, even though the meningothelial subtype was the most predominant (69.8%). The absence of HER2 expression in our study precluded statistical analysis of its relation with other immunomarkers. In the literature, HER2 immunoreactivity in grade I meningiomas shows broad discrepancies, ranging from 8.33 to 87.5% (Table 13). The absence of HER2 expression in our study may be due to changes in the immunomarker or the technique used (TMA).

**Table 13:** Series of meningiomas and respective percentages of HER2 expression.

**Authors, year, country**	**HER2 %**	**Observations**
Current paper	0	96 grade I
Potti et al., 2004, USA (115)	2.35	In grades I, II, III. No relation to prognosis
Loussouarn et al., 2006, France (113)	29.4 grade I	17 grade I, 26.6% in 18 grades II/III. HER2+ > HER2– in recurrent tumors
Kandemir et al., 2010, Turkey (71)	66.0 grade I	53 grade I, mainly in meningothelial tumors. Intense immunostaining in 22.6%. A positive correlation between HER2 and PR+
Abdelzaher et al., 2011, Egypt (51)	8.33 grade I	60 grade I. There was an inverse relation between HER2 and PR expressions, and a positive relation between HER2 and Ki67 expressions
Mahzouni and Movahedipour, 2012, Iran (42)	38.5 grade I	52 grade I, 55.5% in grades II/III
Ongaratti et al., 2016, Brazil (53)	87.5 grade I	48 grade I; 91.6% in 12 grades II/III, (*p* > 0.05)
Telugu et al., 2016, India (4)	75.0 grade I	80 grade I, 18 grade II (HER2, 72.2%) and 2 grade III (HER2, 0%)
Arnli et al., 2018, Norway (57)	48.0	129 grade I, 56 grade II and 1 grade III
Faisal et al., 2019, Indonesia (7)	25.0 grade I	26 grade I; 4 grade II, 18.7%; 3 grade III, 9.37% (*p* > 0.05)

Some studies have shown higher HER2 expressions in grade I meningiomas (116, 117), while other studies have shown no significant differences between grades (7, 42, 53, 113, 115, 118). In some studies, the relation between HER2 expression and an increase in Ki67 and recurrence was evidenced, indicating that they may be predictors of aggression (51, 119). However, other works indicate no relation between positive HER2 expression when correlated with primary and recurrent meningiomas (42, 53). There are, therefore, numerous discrepancies regarding the action of HER2 in relation to the biological behavior of meningiomas, and these suggest HER2 does not participate in aggressiveness.

## Study Limitation

The immunomarkers were studied in 1 mm TMA, which may lead to tumor sampling errors. The absence or low expression of certain markers in this study could be due to the low representativity of the lesion by TMA. Further research will evaluate these same markers in the donor block of the samples analyzed here by TMA.

The recurrence and regrowth were included in the same group, which could be considered an analysis bias.

## Conclusions

Tumor size was directly related to calcifications and peritumoral edema. Therefore, an increase on the disease-free survival is associated with larger extension of resection (Simpson I/II). Cell proliferation marker (Ki67) appears to be a good marker for recurrence/regrowth in grade I meningiomas. No relation was determined between the HLA-E, PR, ER, AR, p53, and COX-2 immunomarkers and recurrence/regrowth in grade I meningiomas.

## Data Availability Statement

All datasets generated for this study are included in the article/Supplementary Material.

## Ethics Statement

This research was approved by the Research Ethics Committee of the Santa Casa de Belo Horizonte Ensino e Pesquisa - EP/SCBH, under protocol CEP 664.402, and only the data of patients who accepted and signed a term of free informed consent were included in the analyses. The patients/participants provided their written informed consent to participate in this study.

## Author Contributions

GCC and WS-M reviewed the clinical data and patient follow-up, performed the literature search, wrote the main text, finalized the manuscript, and conceived the ideas. KM processed the surgical specimens and formatted the clinical and laboratory data. CN accomplished the ki67. AS performed the statistical analyzes. LT reviewed the anatomic pathology slides and carried out others immunomarkers analyzes. RS supervised and reviewed the entire study. All authors contributed to the article and approved the submitted version.

## Conflict of Interest

The authors declare that the research was conducted in the absence of any commercial or financial relationships that could be construed as a potential conflict of interest.
