# Supplementary material for: Recurrence/Regrowth in Grade I Meningioma: How to Predict?
Source: Front Oncol. 2020 Aug 4;10:1144. doi: 10.3389/fonc.2020.01144 (PMC7438949; doi:10.3389/fonc.2020.01144)
Supplement: Supplementary file 1 [file Data_Sheet_1.pdf]

Supplementary Material

Table S1:1 Relation between Ki67 and epidemiological variables, immunomarkers, imaging, histopathology and clinical evolution.

**Variable**		**Ki67**									
		**n**	**Mean**	**Standard deviation**	**Median**	**P 25**	**P 75**	**Min.**	**Max.**	**p value**
**Sex**	Female	69	2.51	2.34	**2.00**	1.40	3.40	0.00	16.00	0.363
	Male	27	2.86	2.14	**2.20**	1.60	4.00	0.20	10.40	
PR	-	35	2.70	2.84	**2.00**	0.80	3.60	0.00	16.00	0.790
	+	57	2.56	1.96	**2.20**	1.40	3.20	0.40	10.40	
ER	-	92	2.58	2.31	**2.10**	1.40	3.40	0.00	16.00	0.626
	+	2	3.20	2.55	**3.20**	1.40	5.00	1.40	5.00	
AR	-	87	2.52	2.33	**1.80**	1.40	3.40	0.00	16.00	0.097
	+	6	3.60	2.33	**2.90**	2.60	4.80	1.40	7.00	
p53	-	7	2.66	1.29	**2.20**	1.60	4.00	0.80	4.20	0.481
	+	88	2.61	2.37	**2.00**	1.40	3.40	0.00	16.00	
COX-2	-	0								
	+	93	2.59	2.32	**2.00**	1.40	3.40	0.00	16.00	
**Calcification**	No	86	2.58	2.38	**2.00**	1.40	3.40	0.00	16.00	0.272
	Yes	10	2.79	1.38	**2.90**	1.80	3.60	0.40	4.80	
**Edema**	No	68	2.56	2.56	**1.80**	1.30	3.30	0.00	16.00	0.120
	Yes	28	2.73	1.45	**2.50**	1.60	3.60	0.40	7.00	
**Recurrence**	No	82	2.45	2.27	**1.80**	1.20	3.40	0.00	16.00	**0.014**
	Yes	14	3.52	2.25	**3.10**	2.00	3.80	1.50	10.40	
**Size**	Medium	27	2.66	2.02	**2.20**	1.40	3.60	0.40	10.40	0.577
	Large	55	2.63	2.61	**2.00**	1.20	3.40	0.00	16.00	

Legend: Ki67, cell proliferation index by MIB-1/Ki67 monoclonal antibody; P, percentile; PR, progesterone receptor; ER, estrogen receptor; AR, androgen receptor; p53, nuclear phosphoprotein p53; COX-2, cyclooxygenase-2; HLA-G, human leukocyte antigen G; HLA-E, human leukocyte antigen E. Note: Mann Whitney test, * Kruskal-Wallis.

Cont. Table S1: Relation between Ki67 and epidemiological variables, immunomarkers, imaging, histopathology and clinical evolution..

Variable		Ki67								
		n	Mean	Standard deviation	Median	P 25	P 75	Min.	Max.	p value
HLA-G	-	0								
	+	90	2.65	2.34	**2.20**	1.40	3.40	0.00	16.00	
HLA-E	-	3	1.00	1.56	**0.20**	0.00	2.80	0.00	2.80	0.105
	+	87	2.69	2.35	**2.20**	1.40	3.60	0.40	16.00	
**Subtypes of grade I meningiomas**	Fibrous	7	1.86	0.96	**2.20**	0.80	2.40	0.20	2.80	0.193
	Meningothelial	67	2.62	2.53	**1.80**	1.40	3.40	0.40	16.00	
	Transitional	14	3.36	1.78	**3.80**	1.60	4.80	0.40	5.60	
**Follow-up**	Alive/death unrelated	75	2.57	2.10	**2.20**	1.40	3.40	0.40	16.00	0.409
	Dead	21	2.73	2.93	**1.80**	0.80	3.40	0.00	10.40	

Legend: Ki67, cell proliferation index by MIB-1/Ki67 monoclonal antibody; P, percentile; PR, progesterone receptor; ER, estrogen receptor; AR, androgen receptor; p53, nuclear phosphoprotein p53; COX-2, cyclooxygenase-2; HLA-G, human leukocyte antigen G; HLA-E, human leukocyte antigen E. Note: Mann Whitney test, * Kruskal-Wallis.

Table S2: Percentage of Ki67 expression in series of meningiomas.

Authors, year, country	No. of cases of grade I meningiomas	Ki67: Mean ± SD (Amplitude)/% [median]	Mean Ki67 in primary tumors [median]	Mean Ki67 in recurrent tumors [median]	p: primary *vs* recurrent tumor correlation
Carvalho et al., 2019, Brazil	96	2.61 ± 2.29(0-16) [median=2.10]	2.45 [1.80]	3.52 [3.10]	Median. p=0.014
Amatya et al., 2001, Japan	109	1.5±1.3 (0 – 72)			
Konstantinidou et al., 2003, Greece	51	1.0 (0.1 – 9.2)			
Wolfsberger et al., 2004, Austria/Germany	55	3.5			
Whittle et al., 2004, Scotland (120)	Review	1.0 – 1.35	0.39 – 3.8	2.3 – 10.9	p < 0.005
Roser et al., 2004, Germany	526	3.54 ± 4.97 (0 – 58)			
Korhonen et al., 2006, Finland	441 primary tumors	3.1 ± 2.8			
Omulecka et al., 2006, Poland	46	3.7			
Maiuri et al., 2007, Italy	100		<	>	P = 0.0001

Cont. Table S2: Percentage of Ki67 expression in series of meningiomas.

Authors, year, country	No. of cases of grade I meningiomas	Ki67: Mean ± SD (Amplitude)/% [median]	Mean of Ki67 in primary tumors [median]	Mean of Ki67 in recurrent tumors [median]	p: primary *vs* recurrent tumor correlation
Takei et al., 2008, USA	46	2.9 ± 2.0 (0.35 – 8.4)			
Abry et al., 2010, Norway	Metanalysis	3.0 (1 – 16)	2.0 (0 – 20)	4.0	p < 0.05
Kandemir et al., 2010, Turkey	53	2.8 ± 2.0			
Kärja et al., 2010, Finland/Sweden	59	(0 – 10)	3.3 ± 0.4	3.9 ± 0.5	p = 0.342
Shayanfar et al., 2010, Iran	63	2.98 ± 2.27			
Abdelzaher et al., 2011, Egypt	60	2.37 ± 1.86			P = 0.002
Babu et al., 2011, India	211	4.07 ± 3.83	<	>	p = 0.0093
Al-Nuamy et al., 2012, Iraq	42	1.8 ± 3.5			
Lee et al., 2013, South Korea	49	2.4 (0 – 18)			
Pavelin, 2013, Croatia	142	1.5 (0 – 13.9)			
Iplikcioglu et al., 2014, Turkey	48	3.34 ± 3.27			
Mukhopadhyay et al., 2017, India	82	2.57 ± 1.67			
